# Supplementary material for: Adult Neurogenesis of the Medial Geniculate Body: In Vitro and Molecular Genetic Analyses Reflect the Neural Stem Cell Capacity of the Rat Auditory Thalamus over Time
Source: Int J Mol Sci. 2024 Feb 23;25(5):2623. doi: 10.3390/ijms25052623 (PMC10932413; doi:10.3390/ijms25052623)
Supplement: Supplementary file 1 [file ijms-25-02623-s001.zip › ijms-2814971 - supplementary.pdf]

International Journal of
Molecular Sciences

MDPI

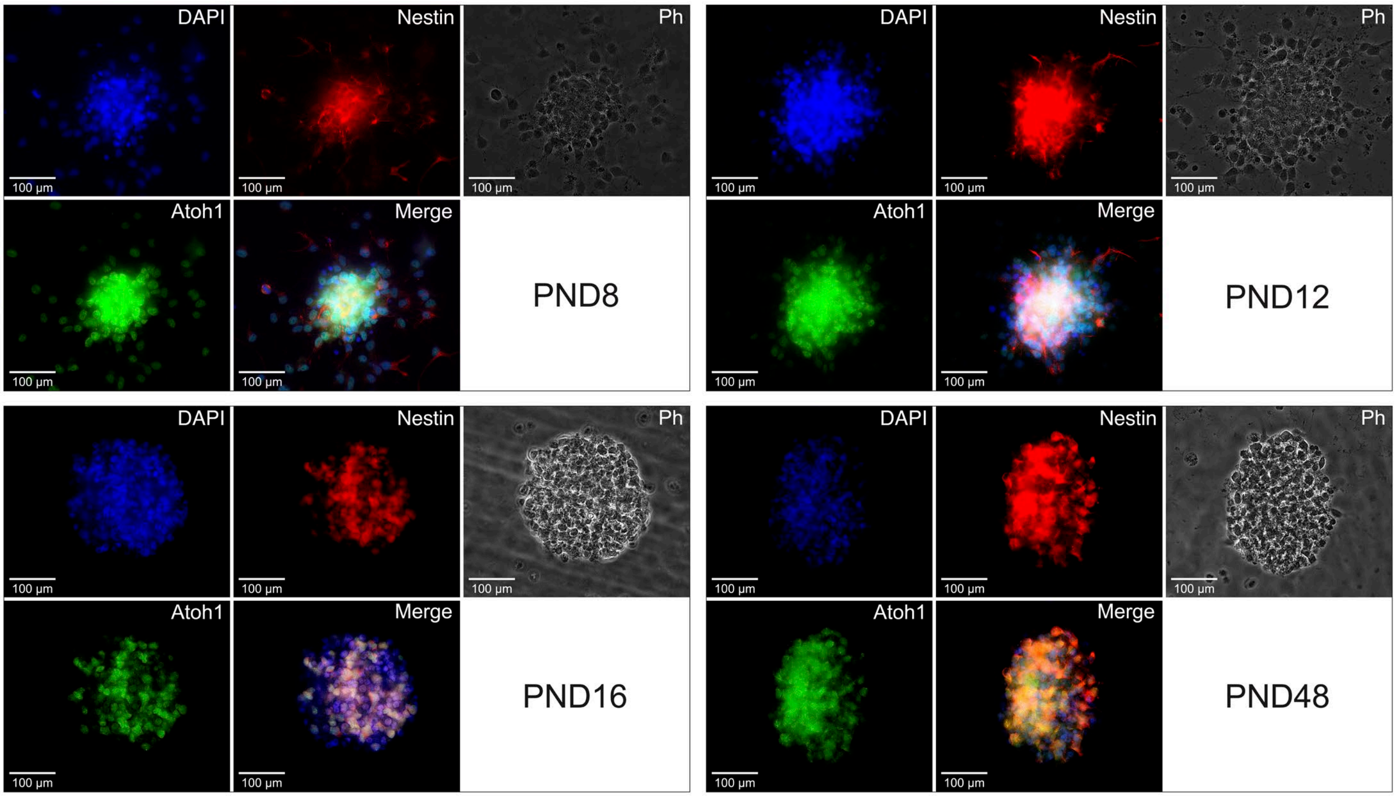

Figure S1. Immunocytological staining of the individual antibodies (DAPI, Nestin, Atoh1) and phase-contrast images of the neurospheres of all examined age groups in addition to Fig. 5 a-d.

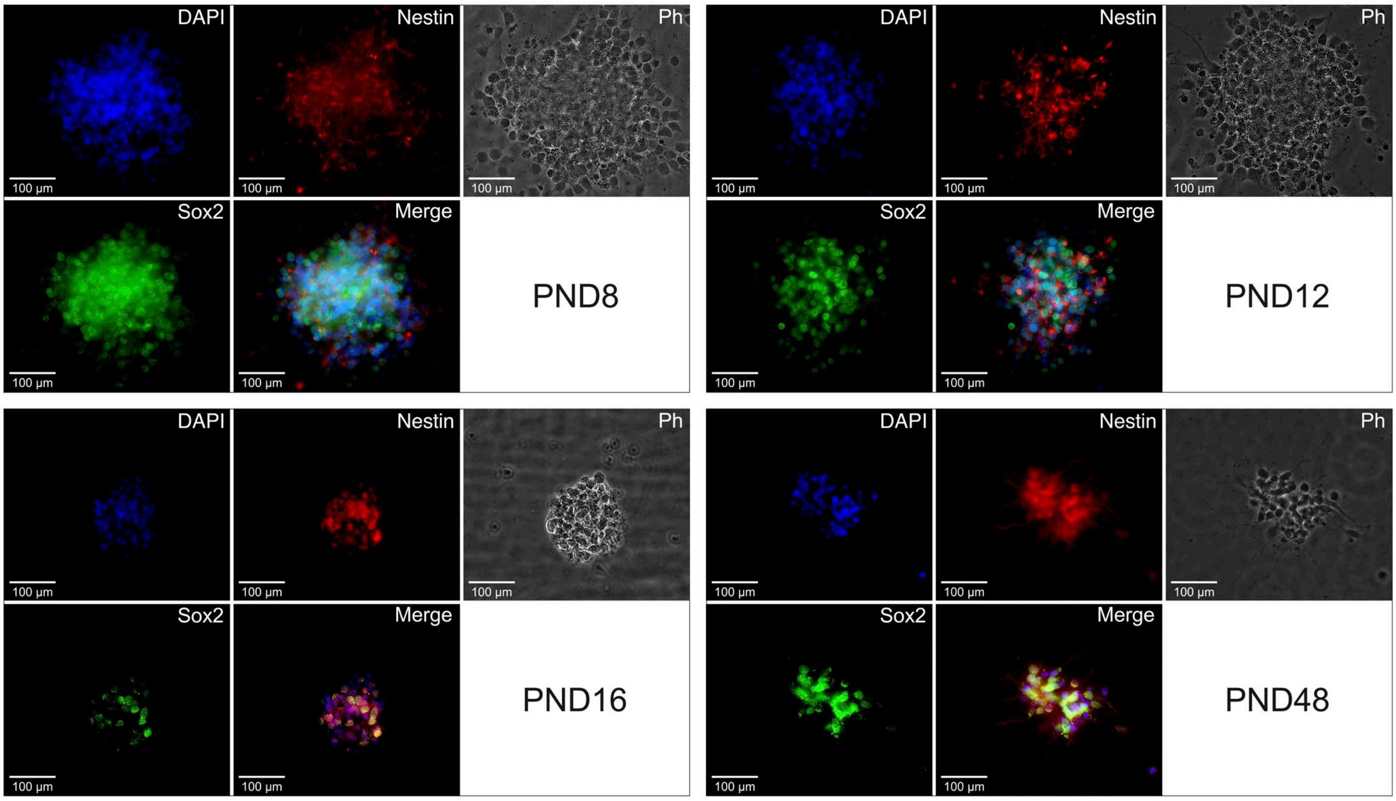

Figure S2. Immunocytological staining of the individual antibodies (DAPI, Nestin, Sox2) and phase-contrast images of the neurospheres of all examined age groups in addition to Fig. 5 e-h.

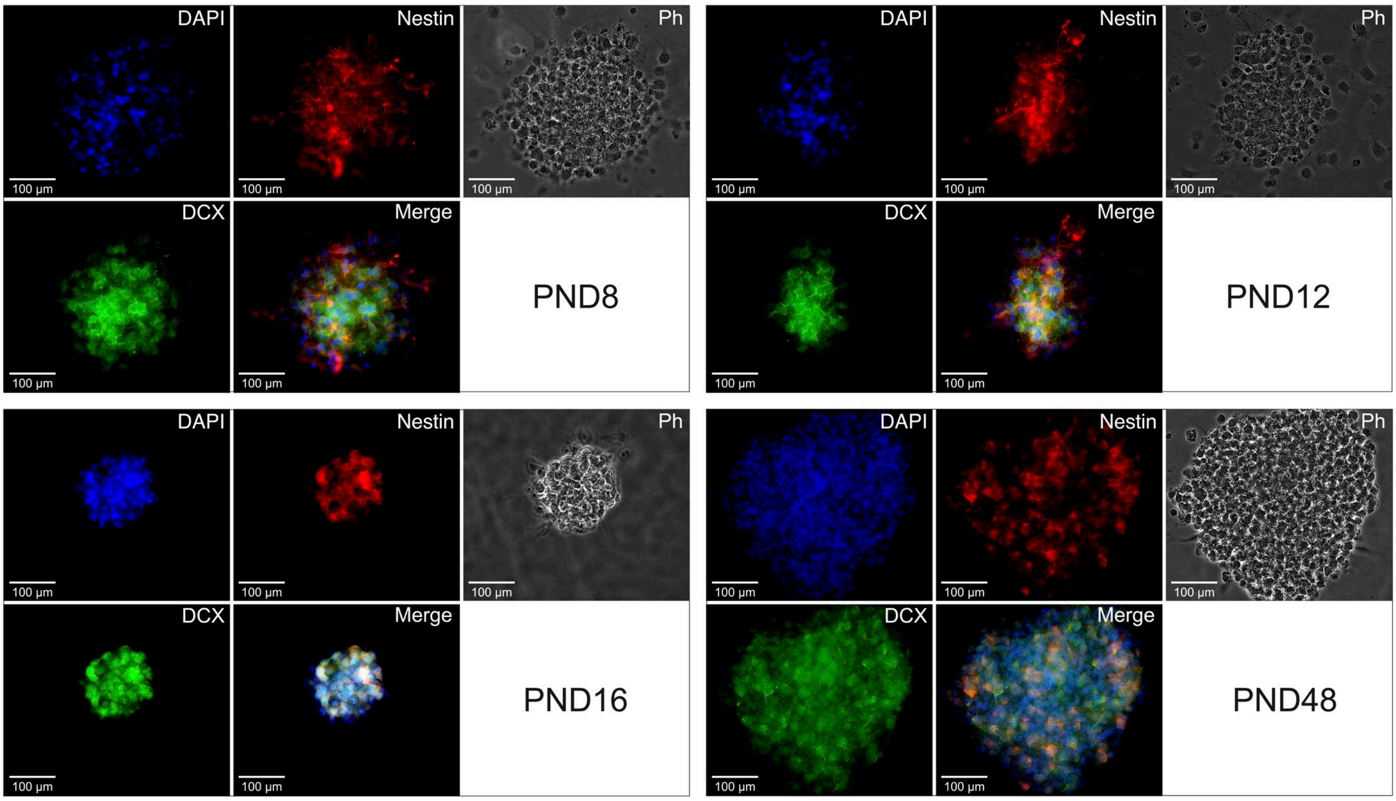

Figure S3. Immunocytological staining of the individual antibodies (DAPI, Nestin, DCX) and phase-contrast images of the neurospheres of all age groups examined in addition to Fig. 5 i-l.
